# SYNCRIP Modulates the Epithelial-Mesenchymal Transition in Hepatocytes and HCC Cells

**DOI:** 10.3390/ijms23020913

**Published:** 2022-01-14

**Authors:** Veronica Riccioni, Flavia Trionfetti, Claudia Montaldo, Sabrina Garbo, Francesco Marocco, Cecilia Battistelli, Alessandra Marchetti, Raffaele Strippoli, Laura Amicone, Carla Cicchini, Marco Tripodi

**Affiliations:** 1 Department of Molecular Medicine, Istituto Pasteur Italia-Fondazione Cenci Bolognetti, Sapienza University of Rome, 00161 Rome, Italy; veronica.riccioni@uniroma1.it (V.R.); flavia.trionfetti@uniroma1.it (F.T.); sabrina.garbo@uniroma1.it (S.G.); francesco.marocco@uniroma1.it (F.M.); cecilia.battistelli@uniroma1.it (C.B.); alessandra.marchetti@uniroma1.it (A.M.); raffaele.strippoli@uniroma1.it (R.S.); laura.amicone@uniroma1.it (L.A.); 2 National Institute for Infectious Diseases L. Spallanzani, IRCCS, 00149 Rome, Italy; claudia.montaldo@inmi.it

**Keywords:** hnRNPQ, EMT, mirRNAs, HCC, RNA binding proteins, metastasis

## Abstract

Heterogeneous nuclear ribonucleoproteins (hnRNPs) control gene expression by acting at multiple levels and are often deregulated in epithelial tumors; however, their roles in the fine regulation of cellular reprogramming, specifically in epithelial–mesenchymal transition (EMT), remain largely unknown. Here, we focused on the hnRNP-Q (also known as SYNCRIP), showing by molecular analysis that in hepatocytes it acts as a “mesenchymal” gene, being induced by TGFβ and modulating the EMT. SYNCRIP silencing limits the induction of the mesenchymal program and maintains the epithelial phenotype. Notably, in HCC invasive cells, SYNCRIP knockdown induces a mesenchymal–epithelial transition (MET), negatively regulating their mesenchymal phenotype and significantly impairing their migratory capacity. In exploring possible molecular mechanisms underlying these observations, we identified a set of miRNAs (i.e., miR-181-a1-3p, miR-181-b1-3p, miR-122-5p, miR-200a-5p, and miR-let7g-5p), previously shown to exert pro- or anti-EMT activities, significantly impacted by SYNCRIP interference during EMT/MET dynamics and gathered insights, suggesting the possible involvement of this RNA binding protein in their transcriptional regulation.

## 1. Introduction

Epithelial–mesenchymal transition (EMT) is a cellular reprogramming mechanism that allows epithelial cells to acquire mesenchymal properties. This transdifferentiation process has a key role in physiology and pathology, being required in the embryo for gastrulation and morphogenesis, in the adult for wound healing, and in epithelial tumors for several functions, such as stemness, resistance to therapy and, mainly, malignant progression. Transitional cells, indeed, can migrate and disseminate, allowing carcinoma cells to metastasize. In secondary sites, mesenchymal cells can reacquire an epithelial phenotype by undergoing mesenchymal–epithelial transition (MET), which is regulated by tumor niche (reviewed in [1]).

Master transcriptional factors (EMT-TFs) (including Slug (Snail2), Twist-related protein 1 (Twist1), zinc-finger E-box-binding homeobox 1 and 2 (Zeb1 and Zeb2), and, primarily, Snail (Snai1), induce the EMT program in a non-redundant manner [2,3,4]. Moreover, a fine crosstalk between EMT-TFs and the involvement of several ncRNAs, including microRNAs (e.g., miR-200 family members [5,6] and long non-coding RNAs (lncRNAs, e.g., HOTAIR [7,8])), have been described. In this complex scenario, the EMT/MET dynamics may also result in metastability, a hybrid state in which epithelial and mesenchymal features are co-expressed [9,10].

Heterogeneous nuclear ribonucleoproteins (hnRNPs) represent a class of RNA Binding Proteins (RBPs), with conserved RNA-binding domains (RBDs), that control different classes of cellular RNAs. HnRNPs, indeed, are involved in translational regulation, alternative splicing, mRNA stabilization, pri-miRNAs processing as well as miRNAs compartmentalization (reviewed in [11]). HnRNPs can also bind pyrimidine-rich DNA sequences, including those at promoters, and are involved in chromatin remodeling and transcription, telomere elongation, and monitoring the genome integrity [12,13,14,15]. Because of their pleiotropic functions, hnRNPs are often deregulated in pathological conditions, particularly in tumors. However, while it is conceivable that hnRNPs can be involved in the fine regulation of cellular reprogramming, their functions in the regulation of EMT is still largely uncharacterized. Current evidence is limited to the regulation of (i) Snail by hnRNP-A2/B1 and hnRNP-F, respectively, in lung [16] and bladder [17] cancer cells, (ii) disabled-2 (Dab2) and interleukin-like EMT inducer (ILEI) by hnRNP-E1 in mammary gland cells [18,19], and, finally, (iii) invasion by PCBP-1 in hepatoma cells [20].

In this work, we specifically focused on the role in the EMT of hnRNP-Q, also known as Synaptotagmin-binding Cytoplasmic RNA-Interacting Protein (SYNCRIP). This evolutionarily conserved RBP recognizes different RNA sequences to modulate different processes such as pre-mRNA splicing, mRNA translation, and pri-miRNA processing [21,22,23,24,25,26,27,28,29,30,31,32]. Moreover, it affects miRNA localization by mediating the partition between the intracellular compartment and extracellular vesicles (EVs) [33,34]. Notably, a role for SYNCRIP in the development and differentiation of specific cell lineages has been described [24,35], as well as its aberrant regulation in different disorders, including cancers [32,36,37,38,39,40]. In particular, SYNCRIP expression represents an unfavorable prognostic marker for hepatocarcinoma (HCC) ([40]; see also https://www.proteinatlas.org, 2 November 2021). The molecular heterogeneity of HCC advanced stages is clearly increased by EMT plasticity, and tumor progression is associated with hepatocyte dedifferentiation and the acquisition of invasive properties [41].

Despite this body of evidence, a link between SYNCRIP overexpression and the ability of cancer cells to metastasize has not yet been clarified.

Here, SYNCRIP upregulation was found to occur during transforming growth factor (TGF)β-induced EMT. The impairment of this hnRNP in hepatocytes interfered with the responsiveness to TGFβ in terms of morphological changes as well as the modulation of epithelial and mesenchymal gene expression. Moreover, in murine invasive HCC cells, SYNCRIP knockdown was demonstrated to impair migration as well as mesenchymal phenotype.

In exploring possible molecular mechanisms underlying these observations, evidence for the ability of SYNCRIP to regulate specific miRNAs (i.e., miR-181-a1-3p, miR-181-b1-3p, miR-122-5p, miR-200a-5p, and miR-let7g-5p), previously known to exert pro- or anti-EMT activities by targeting EMT-TFs (i.e., Snail and Zeb2) [5,42,43,44,45,46,47,48,49,50,51], was gathered in both cell models. Furthermore, the provided data suggested the involvement of SYNCRIP in the transcriptional regulation of these miRNAs during EMT/MET dynamic.

## 2. Results

### 2.1. SYNCRIP Is Involved in TGFβ-Induced EMT of Hepatocytes

To explore the possible SYNCRIP function in the EMTs, we treated non-tumorigenic hepatocytes with a TGFβ cytokine previously found to be sufficient to induce Snail expression in these cells and the loss of the epithelial differentiated phenotype [8,52].

**Figure 1:**
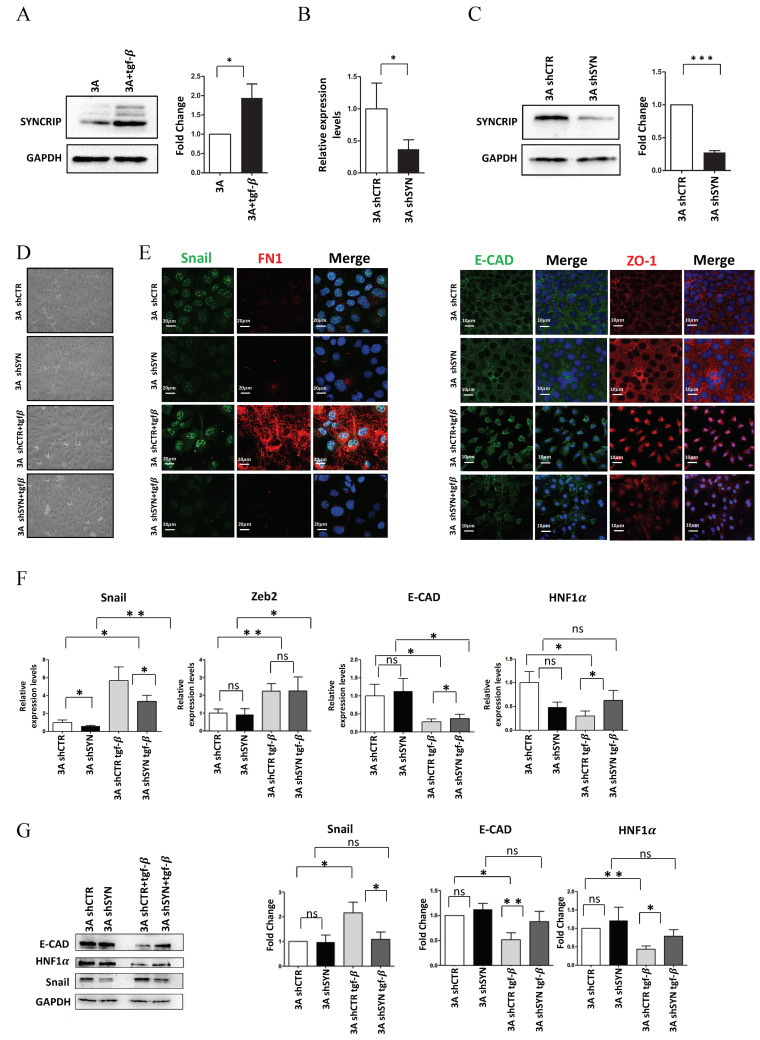
SYNCRIP is involved in TGFβ-induced EMT of hepatocytes. (**A**) Western blot analysis of intracellular levels of SYNCRIP in hepatocytes (3A) treated or not for 24 h with TGFβ. GAPDH was used as loading control. All the experiments were performed three times and images are representative of one indicative experiment of the independent ones. The densitometry analysis (right panel) was conducted by using Image J software. Data are reported as means ± SD. Statistically significant differences are reported (* *p* < 0.05). (**B**) qRT-PCR analysis of *Syncrip* intracellular levels in hepatocytes stably silenced for SYNCRIP (3A shSYN) compared to cells with the empty vector (3A shCTR). The values are calculated via the 2 (−ΔCt) method, normalized to the 18S ribosomal RNA levels and shown as mean ± SD. Statistically significant differences are reported for four independent experiments (* *p <* 0.05). (**C**) Western blot analysis of SYNCRIP and GAPDH as loading control. The image is representative of four independent experiments. The densitometry analysis (right panel) was conducted by using the Image J software. Data are means ± SD of four independent experiments. Statistically significant differences are reported (*** *p <* 0.001). (**D**) Phase contrast micrographs of 3A shSYN cells or 3A shCTR, treated or not for 24 h with TGFβ as indicated. (**E**) Immunofluorescence assays for epithelial (ZO-1 and E-Cadherin) and mesenchymal (SNAIL and FIBRONECTIN) markers in TGFβ-treated or untreated 3A shCTR and 3A shSYN cells. Nuclei were stained with Hoechst (blue). Images are representative of three independent experiments. Scale bar is indicated. (**F**) qRT-PCR analysis for the indicated mesenchymal (*Snail* and *Zeb2*) and epithelial (*E-cadherin* and *HNF1α*) markers in TGFβ-treated or untreated 3A shCTR and 3A shSYN cells. The values are calculated by the 2 (−ΔCt) method, normalized to the 18S ribosomal RNA levels and shown as mean ±  SD. Statistically significant differences are reported for five independent experiments (* *p <* 0.05; ** *p <* 0.01; ns = no significance). (**G**) Western blot analysis of intracellular levels of the indicated epithelial and mesenchymal markers in TGFβ-treated or untreated 3A shCTR and 3A shSYN cells. The image is representative of four independent experiments. GAPDH was used as loading control. The densitometry analysis (right panel) was conducted by using the Image J software. Data are means ± SD of four independent experiments. Statistically significant differences are reported (* *p <* 0.05; ** *p <* 0.01; ns = no significance).

As shown in Figure 1A, the analysis of SYNCRIP levels highlighted that this RBP was upregulated in the transdifferentiation process. In order to evaluate whether SYNCRIP modulation was only correlative or rather causal to the EMT, we analyzed the effects of SYNCRIP knockdown. To achieve this aim, hepatocytes were stably infected with retroviral vectors expressing different shRNAs against SYNCRIP (shSYN) and a scrambled sequence as a control (shCTR). RT-qPCR and Western blot analysis validated that SYNCRIP levels in shSYN cells were significantly reduced by the viral vectors targeting its transcript (Figure 1B,C; in line with [33]).

As shown in Figure 1D,E, morphological and immunofluorescence analysis in TGFβ-treated shCTR hepatocytes confirmed the occurrence of EMT by a morphological change from an epithelial, cobblestone-like phenotype to a more spindle-shaped mesenchymal phenotype with (i) Snail and fibronectin positive regulation and (ii) delocalization of the epithelial markers ZO-1 and E-Cadherin. Conversely, SYNCRIP-interfered cells retained a more epithelial morphology after EMT induction, with the partial retention of ZO-1 and E-Cadherin in the membrane and undetectable Snail and fibronectin expression. Coherently, qRT-PCR (Figure 1F) and WB (Figure 1G) analysis showed that while the SYNCRIP-knockdown did not interfere with the TGFβ-mediated upregulation of the EMT-TF Zeb2, it significantly prevented the induction of Snail protein and the downregulation of its main epithelial target genes (E-cadherin and HNF1α).

Overall, these findings indicate that SYNCRIP can act as a mesenchymal gene, positively regulated in TGFβ-induced EMT, and provide evidence of a functional role for this hnRNP in the transdifferentiation of the hepatocytes by contributing to the modulation of Snail and its mediated gene regulation.

### 2.2. SYNCRIP Impairment Affects Mesenchymal Phenotype and Migratory Properties of HCC Cells

**Figure 2:**
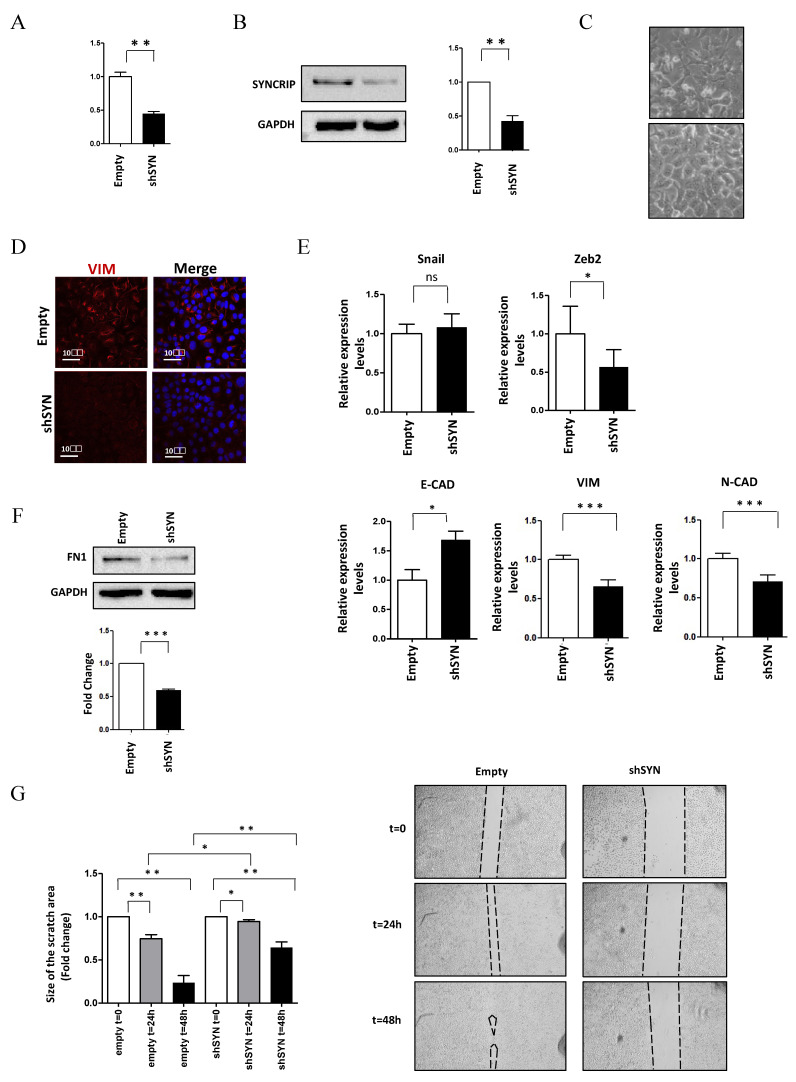
SYNCRIP impairment affects mesenchymal phenotype and migratory properties of HCC cells. (**A**) qRT-PCR analysis of *Syncrip* intracellular levels in BW1J hepatoma cells silenced for SYNCRIP (shSYN) compared to cells transfected with the empty vector (empty). The values are calculated by the 2 (−ΔCt) method, normalized to the 18S ribosomal RNA levels, and shown as mean ± SD. Statistically significant differences are reported for five independent experiments (** *p <* 0.01). (**B**) Western blot analysis of SYNCRIP and GAPDH as loading control. The image is representative of three independent experiments. The densitometry analysis (right panel) was conducted by using the Image J software. Data are means ± SD of three independent experiments. Statistically significant differences are reported (** *p <* 0.01). (**C**) Phase contrast micrographs of BW1J shSYN and BW1J empty. (**D**) Immunofluorescence assays for the mesenchymal marker VIMENTIN in BW1J shSYN and BW1J empty. Nuclei were stained with Hoechst (blue). Images are representative of three independent experiments. Scale bar, 10 μm. (**E**) qRT-PCR analysis of intracellular levels of *E-cadherin* and of the mesenchymal genes *Zeb2*, *Snail*, *Vimentin*, and *N-cadherin* in BW1J shSYN and BW1J empty. The values are calculated by the 2 (−ΔCt) method, normalized to the 18S ribosomal RNA levels and shown as mean ± SD. Statistically significant differences are reported for six independent experiments (* *p <* 0.05; *** *p <* 0.001; ns = no significance). (**F**) Western blot analysis of intracellular levels of the mesenchymal marker Fibronectin (FN1) in BW1J shSYN and BW1J empty. GAPDH was used as loading control. The densitometry analysis was conducted by using the Image J software. Data are means ± SD of three independent experiments. Statistically significant differences are reported (*** *p <* 0.001). (**G**) Scratch assay of BW1J shSYN compared to control cells at the indicated time. Quantification was performed using the Fiji-ImageJ image processing package. Statistically significant differences are reported for four independent experiments (* *p <* 0.05; ** *p <* 0.01).

To shed light on the functional role of SYNCRIP in invasive HCC cells, the effects of its knockdown were further assessed in murine hepatoma mesenchymal-like BW1J cells. As shown in Figure 2, SYNCRIP impairment in BW1J cells (Figure 2A,B) allowed a MET with the rescue of a more differentiated phenotype. Morphological (Figure 2C) and molecular analysis (Figure 2D,E,F), indeed, highlighted the strong negative regulation of the mesenchymal markers Vimentin and Fibronectin, the downregulation of the EMT-TF Zeb2 and N-cadherin with the positive modulation of E-cadherin levels.

Notably, SYNCRIP was found to be involved in the acquisition of the migratory ability of HCC cells, as demonstrated by scratch assays of BW1J cells in which SYNCRIP knockdown significantly reduced cell motility (Figure 2G). Overall, these data demonstrate a positive role for SYNCRIP in allowing migration as well as in the maintenance of the mesenchymal phenotype of HCC cells.

### 2.3. SYNCRIP Controls Anti- and Pro-EMT miRNA Levels

In order to gain insight into the mechanism of SYNCRIP function in the acquisition of mesenchymal properties by epithelial cells, we focused on specific miRNAs (i.e., miR-122-5p, miR-200a-5p, miR-let7g-5p, miR-181a1-3p and miR-181b1-3p), expressed by the hepatocytes and broadly involved in the EMT of different cell types [5,33]: notably, miR-122-5p and miR-200a-5p are well known to inhibit different EMT-TFs [5,42,43,44,45,46], while miR-181a1-3p and miR-181b1-3p act as metastasis-promoting factors leading to Snail stabilization [47,48].

**Figure 3:**
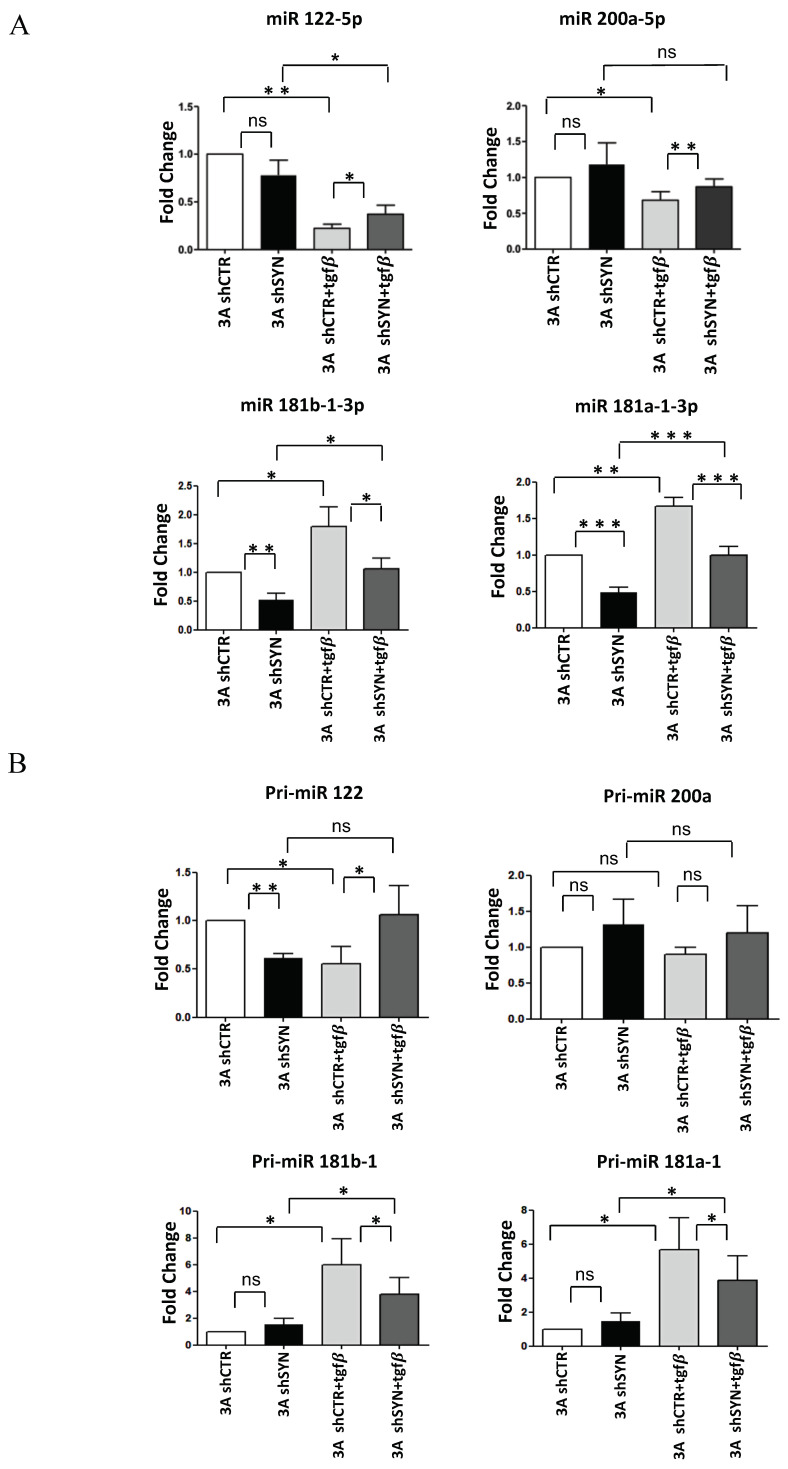
SYNCRIP controls anti and pro-EMT miRNA levels in hepatocytes undergoing TGFβ-induced EMT. (**A**) qRT-PCR analysis of intracellular levels of anti-EMT microRNAs (*miR-122-5p* and *miR-200a-5p*) and pro-EMT microRNAs (*miR-181a1-3p* and *miR-181b1-3p*) in 3A shCTR and 3AshSYN cells treated or not with TGFβ. The values are calculated via the 2 (−ΔCt) method, normalized to the cel-miR-39, expressed as fold enrichment, and shown as mean ± SD. Statistically significant differences are reported for six independent experiments (* *p <* 0.05; ** *p <* 0.01; *** *p <* 0.001; ns = no significance). (**B**) qRT-PCR analysis of intracellular levels of the indicated pri-miRNAs in the same cells as in (**A**). The values are calculated via the 2 (−ΔCt) method, normalized to the 18S ribosomal RNA levels, and shown as mean ± SD. Statistically significant differences are reported for four independent experiments (* *p <* 0.05; ** *p <* 0.01; ns = no significance).

Firstly, we monitored mature miRNAs levels in hepatocytes undergoing EMT. As shown in Figure 3A, RT-qPCR analysis demonstrated that the expected TGFβ repression of miR-200a-5p and miR-122-5p, and induction of miR-181a1-3p and miR-181b1-3p were significantly interfered with by SYNCRIP knockdown. Then, to evaluate at which level of control of miRNAs regulation SYNCRIP could participate, the expression levels of primary miRNAs (pri-miRs) transcripts, whose processing generates mature miRs, were further investigated. As shown in Figure 3B, in both silenced and control hepatocytes undergoing EMT, the induction of pri-miR-181a1, pri-miR-181b1, and the repression of pri-miR-122 matched that of the respective miRs. The same modulation of the corresponding mature form, even if not significant, was observed for pri-miR-200a. These data indicate that SYNCRIP impacts the TGFβ-mediated regulation of specific miRs and, as its effect was detectable at the pri-miR level, suggest that this regulation may occur at the transcriptional level.

**Figure 4:**
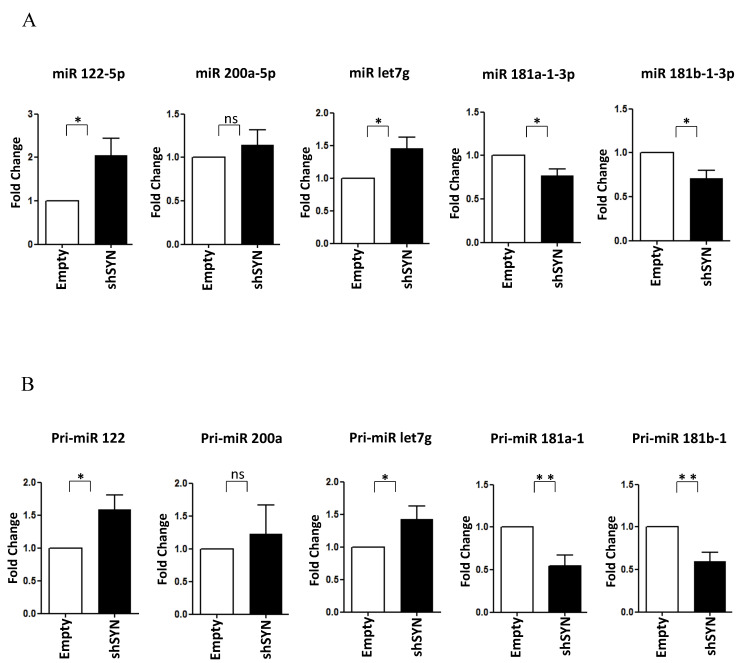
SYNCRIP controls anti and pro-EMT miRNA levels in hepatoma cells. (**A**) qRT-PCR analysis of intracellular levels of anti-EMT microRNAs (*miR-122-5p*, *miR-200a-5p*, *and let7-g-5p*) and pro-EMT microRNAs (*miR-181a1-3p and miR-181b1-3p*) in BW1J shSYN compared to control cells (empty). The values are calculated via the 2 (−ΔCt) method, normalized to the cel-miR-39, expressed as fold enrichment, and shown as mean ± SD. Statistically significant differences are reported for four independent experiments (* *p <* 0.05; ns = no significance). (**B**) qRT-PCR analysis of intracellular levels of the indicated pri-miRNAs in the same cells as in (**A**). The values are calculated via the 2 (−ΔCt) method, normalized to the 18S ribosomal RNA levels, and shown as mean ± SD. Statistically significant differences are reported for seven independent experiments (* *p <* 0.05; ** *p <* 0.01; ns = no significance).

Of note, as shown in Figure 4A,B, the SYNCRIP influence on miR-181a1-3p, miR-181b1-3p, and miR-122-5p levels was further confirmed in BW1J cells. Interestingly, miR-let7g-5p, another anti-EMT regulator [49,50,51], as found modulated limitedly to hepatoma cells (Figure 4A and data not shown). Coherently, in BW1J cells modulations of pri-miRs-181, pri-miR-122, and pri-miR-let7g were strictly correlated to that of the corresponding mature forms. Overall, these data indicate that SYNCRIP exhibits a function in miRNA regulation in both the induction (i.e., during EMT) and the maintenance (i.e., in transformed invasive cells) of the mesenchymal state of transdifferentiated hepatocytes.

## 3. Discussion

The main finding of this work was to ascribe a role to the hnRNP-Q, also known as SYNCRIP, in the modulation of EMT/MET dynamics. This RNA-binding protein was found, indeed, as positively regulated in non-tumorigenic hepatocytes by TGFβ and, notably, its impairment prevented the full transdifferentiation. Moreover, SYNCRIP knockdown in HCC invasive cells allowed the rescue of a more differentiated phenotype by MET.

As pleiotropic regulators of gene expression, and often deregulated in epithelial cancers (reviewed in [11]), hnRNPs are conceivably implicated in mediating EMT reprogramming, but their role in this context remains largely unexplored. Here, we demonstrated for the first time, to our knowledge, a direct correlation between the function of SYNCRIP and the EMT outcome. Specifically, SYNCRIP-interfered cells undergoing TGFβ-induced EMT showed a limited Snail induction, in turn correlated to a minor downregulation of its main epithelial targets, E-cadherin and HNF1α, controlling hepatocyte differentiation and the maintenance of the epithelial phenotype [53,54]. These observations were corroborated by functional data in HCC invasive cells, where SYNCRIP knockdown induced MET and significantly impaired their migratory capacity. This evidence provides a possible molecular link between the well-known SYNCRIP overexpression in HCC [40] and the ability of cells to metastasize.

Mechanistically, our results demonstrated that SYNCRIP knockdown in hepatocytes impacted the TGFβ-mediated modulation of specific miRNAs, i.e., miR-122-5p, miR-181-a1-3p, miR-181-b1-3p, and miR-200a-5p. Coherently, SYNCRIP silencing in hepatoma cells modulated miR-122-5p, miR-181-a1-3p, and miR-181-b1-3p, allowing the rescue of the epithelial phenotype, and also determined let-7g-5p upregulation. Our data are in line with previous evidence highlighting a key role for these specific miRNAs as key regulators of EMT and cancer metastasis by orchestrating fine changes in gene expression: (i) miRs-200 acts as an anti-EMT player, mainly inhibiting EMT-TFs (e.g., Snail and Zeb2) [5,43,44,45,46,55], (ii) miR-122-5p is a tissue-specific miRNA [56], highly expressed in the liver, known to target Snail 1 and Snail 2 and whose downregulation has been associated with HCC invasion and metastasis [42]; (iii) let-7g, via the K-Ras/HMGA2/Snail axis, is sufficient to inhibit the proliferation and migration of HCC cells [49]. Conversely, (iv) miR-181 is an EMT-promoting regulator causing Snail protein stabilization [47].

Interestingly, we observed that SYNCRIP knockdown, in TGFβ-treated hepatocytes as well as hepatoma cells, leads to a significant modulation of both the pri and mature forms of the specific miRNAs investigated, thus indicating the conceivable involvement of this hnRNP in their transcription. Even if, to our knowledge, there is no previous evidence of a possible function of SYNCRIP as a transcriptional regulator, the here provided observations suggest, for SYNCRIP, a role in the chromatin context that might extend the function of SYNCRIP in the regulation of miRNAs. It is previously known, indeed, that SYNCRIP mediates (i) their partition between intracellular and extracellular compartments by binding the hEXO motif (GGCU/A) [33,34]; (ii) the processing of let-7a by recognizing the UAGAAU sequence on the apical loop of the correspondent pri-miRNA [22].

Further studies are required to clarify whether the here suggested transcriptional *trans* activation of specific miRNAs by SYNCRIP can require the direct binding of this hnRNP to specific DNA sequences, or if it can be indirect, implying its association with multiprotein complexes of transcriptional regulators and/or epigenetic modifiers or a possible SYNCRIP-mediated control of their synthesis. Note that other hnRNPs, such as hnRNP-U and hnRNP-K, were shown to bind to chromosomal DNA [57,58]. Furthermore, hnRNP-U interacts with p300, thus controlling the hyperacetylation of histones [59], while hnRNP-K can recruit the chromatin remodeling enzyme histone methyltransferase [60] and acts as a transcriptional factor [12,13]. Moreover, given that the role of miRNAs in the fine-tuning of EMT/MET dynamics is not limited to the hepatocytes (e.g., miR-200 family regulation is involved in the progression of different epithelial tumors, including breast and colorectal cancer [46,61,62]), the impact of SYNCRIP in their regulation could also have relevance in other cell types.

Interestingly, SYNCRIP transcription, promoted by the lncNT5E, has a role in pancreatic cancer progression [39].

Further studies are required to dissect whether the function of SYNCRIP in modulating the EMT correlates to the acquisition of drug resistance by HCC cells. This in line with the role of other hnRNPs in chemoresistance [63,64,65] and the observation that the SYNCRIP target miR-200a-3p affects the drug resistance of Hep3B cells [66]. On the other hand, SYNCRIP could potentially be involved in molecular pathways known to be the target of pharmacological approaches [67].

In conclusion, we believe that the major conceptual advance implied by our results is that SYNCRIP represents a new modulator of EMT. Moreover, our data point to the involvement of this regulator in the TGFβ-mediated transcription of specific miRNAs. The further understanding of the mechanism by which SYNCRIP acts will hopefully be instrumental for research on hepatocyte reprogramming and tumorigenesis.

## 4. Materials and Methods

### 4.1. Cell Cultures

Non-tumorigenic murine 3A hepatocytes [68] were grown in RPMI 1640 medium with 10% FBS (GIBCO^®^ Life Technology, Monza, Italy), 50 ng/mL epidermal growth factor (EGF), 30 ng/mL insulin growth factor (IGF-II) (PeproTech Inc., Rocky Hill, NJ, USA), 10 mg/mL insulin (Roche, Mannheim, Germany), and penicillin/streptomycin on dishes coated with collagen I (Collagen I, Rat Tail; Gibco Life Technology, Monza, Italy). When reported, cells were treated with 2,5 ng/mL TGFβ1 (PeproTech Inc., Rocky Hill, NJ, USA) for 24 h. Murine BW1J cells [8] were grown in DMEM supplemented with 10% FBS and penicillin/streptomycin.

### 4.2. SYNCRIP Knockdown

Stable SYNCRIP knockdown was achieved through the infection of 3A cells with pSUPER retroviral constructs (Oligoengine, Seattle, WA, USA) expressing different shRNAs against SYNCRIP and a scrambled sequence as a control (as reported in [33]). Viral supernatants were collected 48 h after the transfection of 293 gp packaging cells, filtered, then added to cells. Selection was performed starting from 48 h post infection with 2 μg/mL puromycin for at least 1 week before successive analysis. To achieve the SYNCRIP knock-down, BW1J cells were transfected with Lipofectamine LTX and Plus reagent (Invitrogen, San Diego, CA, USA) by using equal amounts of the pSUPER shSYNCRIP constructs. Analyses of RNAs and proteins were performed 48 and 72 h after transient transfection, respectively.

### 4.3. RNA Extraction, RT-PCR, and Real-Time qPCR

Total RNA from cells was isolated using Qiazol and the miRNeasy Mini Kit (QIAGEN, Hilden, Germany) following the manufacturer’s protocol. RNA purity was assessed using a spectrophotometric measure of optical density 260 (OD260)/OD280~2 and OD260/OD230 > 1.8 with the Nanodrop 2000c Spectrophotometer (Thermo-Fisher Scientific, Waltham, MA, USA). Total RNA and pri-miRNAs (500 ng) were reverse transcribed with the iScriptTM c-DNA Synthesis Kit (Bio-Rad Laboratories, Hercules, CA, USA). MicroRNAs (150 ng) were reverse transcribed with the MystiCQ cDNA Synthesis Mix (Sigma-Aldrich, St. Louis, MO, USA) according to the manufacturer’s protocol. Diluted cDNA samples were used for qPCR in a total volume of 10 μL using GoTaq qPCR Master Mix (Promega, Madison, WI, USA) and the reactions were carried out in a Bio-Rad-iQ-iCycler. Relative amounts of mRNAs and pri-miRNAs, obtained using the 2 (−ΔCt) method, were normalized with respect to the housekeeping gene 18S rRNA. Relative amounts of miRNAs, obtained using the 2 (−ΔCt) method, were normalized with respect to the synthetic cel-miR-39 (NORGEN, Thorold, ON, Canada). The same amount of cel-miR-39 was added to 150 ng of miRNAs of each sample before reverse transcription and was used as an internal control in Real-Time qPCR. Primer sequences are reported in Table 1.

### 4.4. Western Blotting

Cells were lysed in RIPA buffer (50mM Tris-HCl pH 7.6, 150 mM NaCl, 0.5% sodium deoxycholate, 0.1% SDS, and 1% NP40), containing freshly added cocktail protease inhibitors (Proteo-Guard EDTA-free, TaKaRa, Kusatsu, Shiga-ken, Japan), and protein concentration was determined using a Protein Assay Dye Reagent (Bio-Rad Laboratories, Hercules, CA, USA). Equal amounts of samples (20 μg in Laemmli Buffer) were separated via SDS-PAGE then transferred to a nitrocellulose membrane (Pure Nitrocellulose Membrane 0.45 μm, Bio-Rad Laboratories, Hercules, CA, USA). Blots, after blocking in 5% non-fat milk in TBS-Tween (10 mM Tris-HCl pH 7.5, 150 mM NaCl, and 0.05% Tween 20), were incubated overnight with the following primary antibodies: α-SYNCRIP (MAB11004, Millipore, Burlington, MA, USA), α-HNF1α (NBP1-33596 Novus Biological, Centennial, CO, USA), α-E-Cadherin (610182 BD transduction laboratories, Franklin Lakes, NJ, USA), α-Snail (L70G2, Cell Signaling Technology, Danvers, MA, USA), α-Fibronectin (F0916 Sigma-Aldrich, St. Louis, MO, USA), and α-GAPDH (MAB-374 Millipore Burlington, MA, USA), used as a loading control. The immune complexes were detected with horseradish peroxidase-conjugated species-specific secondary antiserum: α-Rabbit 172-1019 and α-Mouse 170-6516 (Bio-Rad Laboratories, Hercules, CA, USA), α-Goat 705-036-147 (Jackson ImmunoResearch, St. Thomas’ Place Cambridgeshire Business Park, Ely, CB7 4EX, UK) then via chemiluminescence reaction (Clarity Max ECL Substrate and Clarity Western ECL Substrate; Bio-Rad Laboratories, Hercules, CA, USA). Densitometric analysis of protein expression was performed by using the Fiji-ImageJ image processing package.

### 4.5. Immunofluorescence Analysis

For immunofluorescence analysis, cells were fixed in 4% paraformaldehyde (for Snail and FN1), methanol (for ZO-1 and E-cadherin) and permeabilized with Triton-X100. After the unspecific binding sites were blocked with BSA 3% in PBS, cells were incubated for 1 h with the following primary antibodies: Snail mouse monoclonal (Cell Signaling Technology, Danvers, MA, USA), Fibronectin rabbit polyclonal (Abcam, Cambridge, UK), Vimentin rabbit monoclonal (ab92547, Abcam, Cambridge, UK); ZO-1 rabbit polyclonal (#61-7300. Invitrogen, San Diego, CA, USA), and E-cadherin mouse monoclonal (BD Biosciences Pharmingen, San Diego, CA, USA). Secondary antibodies (goat-anti mouse Alexa Fluor 488, Cy3-conjugated secondary antibody, and DRAQ5 staining solution) were from Life Technologies by Thermo Fisher Scientific, Waltham, MA, USA; Jackson Immuno-research, St. Thomas’ Place Cambridgeshire Business Park, Ely, CB7 4EX, UK; and Miltenyi Biotec, Bergisch Gladbach, Germany, respectively. Preparations were examined under a confocal microscope (Leica TCS SP2) with an objective 63X and a CCD camera (Nikon Inc., Japan). Digital images were processed with Adobe Photoshop 7 software (Adobe Systems). The same enhanced color levels were applied for all channels.

### 4.6. Scratch Assay

BW1J cells, transfected with pSUPER shSYNCRIP constructs (Oligoengine, Seattle, WA, USA) or with empty vector (as above), were maintained in culture medium to reach 100% confluence, then shifted to serum-depleted culture medium to inhibit cell proliferation (as reported in [69]). A scratch wound was created on the cell layer using a micropipette tip, and micrographs were taken at 0.24 and 48 h after the scratch. Cell-devoid areas at times of 0.24 and 48 h after the scratch were quantified through the Fiji-ImageJ image processing package.

### 4.7. Statistical Analysis

For qRT-PCR, Western blotting, and scratch assay, statistical differences were assessed with the one-tailed paired Student’s *t*-test using GraphPad Prism Version 6 (GraphPad Software). A *p*-value *p <* 0.05 was considered statistically significant (* *p* < 0.05; ** *p* < 0.01 and *** *p* < 0.001). Data were obtained from independent experiments and expressed as means ± s.e.m.

## Figures and Tables

**Table 1:** Primers used for qPCR analysis.

Gene Name	Primer Sequence
SYNCRIP	For ACCTTGCCAACACGTAACA Rev CCATAGCCTTGACACACCA
Snail	For CCACTGCAACCGTGCTTTT Rev CACATCCGATGGGTTTGG
E-cadherin	For CTACTGTTTCTACGGAGGAG Rev CTCAAATCAAAGTCCTGGTC
HNF1α	For TATCATGGCCTCGCTACCTG Rev ACTCCCCATGCTGTTGATGA
Vimentin	For AGCAGTATGAAAGCGTGGCT Rev CTCCAGGGACTCGTTAGTGC
N-cadherin	For GTGGAGGCTTCTGGTGAAAT Rev CTGCTGGCTCGCTGCTT
18S	For ACGACCCATTCGAACGTCTG Rev GCACGGCGACTACCATCG
mmu-pri-mir-122	For GCTGTGGAGTGTGACAATGG Rev GAGTGGACGGATTGCCTAGC
mmu-pri-mir-let7g	For CGCTCCGTTCTCTTTTGCC Rev CTCCTGTACCGGGTGGTATC
mmu-pri-mir-200a	For GGCCTCTGTGGGCATCTTAC Rev GGTGGGTCACCTTTGAACAT
mmu-pri-mir-181a-1	For CACATCTCTGCCTCACAGGT Rev AGGGTACAATCAACGGTCG
mmu-pri-mir-181b-1	For ATTCATTGCTGTCGGTGGGT Rev AAAAAGCGGGGCCACAGTTG
mmu-miR-122-5p	TGGATGTGACAATGGTGTTTG
mmu-miR-let7g-5p	TGAGGTAGTAGTTGTACAGTT
mmu-miR-200a-5p	CATCTTACCGGACATGCTGGA
mmu-miR-181a1-3p	ACCATCGACCGTGATTGTACC
mmu-miR-181b1-3p	CTCACTGAACAATGAATGC
